# Clinical signs and symptoms associated with WHO severe dengue classification: a systematic review and meta-analysis

**DOI:** 10.1080/22221751.2021.1935327

**Published:** 2021-06-11

**Authors:** Tha Pyai Htun, Zhonghui Xiong, Junxiong Pang

**Affiliations:** a Saw Swee Hock School of Public Health, National University of Singapore and National University Health System, Singapore, Singapore; b Yong Loo Lin School of Medicine, National University of Singapore and National University Health System, Singapore, Singapore

**Keywords:** Severe dengue, signs, symptoms, predictive performance, meta-analysis

## Abstract

The World Health Organization (WHO) introduced the new dengue classification in 2009. We aimed to assess the association of clinical signs and symptoms with WHO severe dengue classification in clinical practice. A systematic literature search was performed using the databases of PubMed, Embase, and Scopus between 2009 and 2018 according to PRISMA guideline. Meta-analysis was performed with the RevMan software. A random or fixed-effect model was applied to pool odds ratios and 95% confidence intervals of important signs and symptoms across studies. Thirty nine articles from 1790 records were included in this review. In our meta-analysis, signs and symptoms associated with higher risk of severe dengue were comorbidity, vomiting, persistent vomiting, abdominal pain or tenderness, pleural effusion, ascites, epistaxis, gum bleeding, GI bleeding, skin bleeding, lethargy or restlessness, hepatomegaly (>2 cm), increased HCT with decreased platelets, shock, dyspnea, impaired consciousness, thrombocytopenia, elevated AST and ALT, gall bladder wall thickening and secondary infection. This review shows new factors comorbidity, epistaxis, GI and skin bleeding, dyspnea, gall bladder wall thickening and secondary infection may be useful to refine the 2009 classification to triage severe dengue patients.

## Introduction

**Figure 1.**
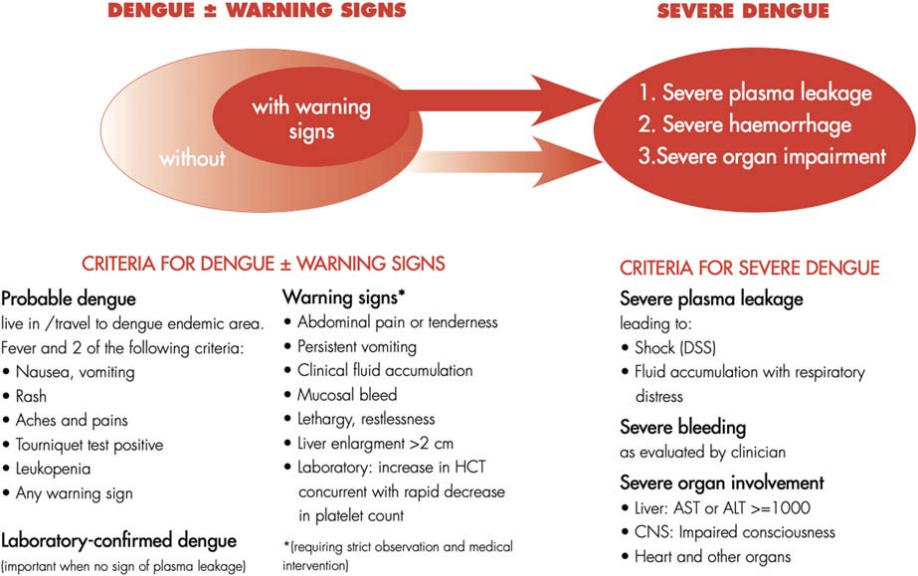
The 2009 WHO revised dengue case classification.

Dengue is the fastest spreading mosquito-borne viral disease globally, affecting 50 million individuals every year [1]. In the vast majority of individuals, dengue fever is a self-limiting disease that requires minimal supportive treatment. However, in less than 1% of patients, symptoms of severe dengue, including clinical fluid accumulation, shock, and multiple organ dysfunction could spell impending demise if left untreated. The new 2009 WHO classification for dengue was hence created to allow clinicians to triage patients easily according to their clinical presentations for more effective clinical management (Figure 1) [1]. This new classification is intended to bring greater clarity on the severity of clinical presentations compared to the 1997 classification of dengue into undifferentiated fever, dengue fever [1] and dengue hemorrhagic fever.

The 1997 classification was proven to underestimate the severity of dengue infection [2]. Multiple studies had shown that plasma leakage causing clinical fluid accumulation, transaminitis and thrombocytopenia were more indicative of severe dengue instead of clinical manifestations of bleeding, as was prioritized in the old classification [3, 4]. In febrile travelers returning from endemic regions, one study showed that a significant number of cases of severe dengue would have been missed if the WHO diagnostic criteria for dengue haemorrhagic fever would have been applied [3]. While many studies have effectively highlighted the shortcomings of the 1997 classification, there is a paucity of studies done today to ascertain if the clinical utility of the current 2009 classification has improved clinical diagnosis and management of dengue infections.

Previous review has reported that the new 2009 classification has a higher sensitivity and specificity compared with the 1997 classification [5]. However, there was a question for applicability in clinical practice and usefulness for triage using the revised dengue classification [6]. Several studies have assessed the association of clinical factors with severe dengue [7–10]. However, risk factors reported among severe dengue patients remained inconsistent [7–10]. The objective of this review was to synthesize the best of available evidence by conducting meta-analysis to assess the factors associated with severe dengue patients. This systematic review and meta-analysis therefore investigate the likelihood of new factors associated with severe dengue, which may be useful to further revise the existing dengue 2009 classifications for more accurate triaging of patients.

## Materials and methods

### Search strategy

This review was conducted according to the standards outlined in the Preferred Reporting Items for Systematic Reviews and Meta-Analyses (PRISMA) statement [11]. No documented review protocol exists for this meta-analysis. The year 2009 was selected as the start date of searching articles as the introduction of new WHO dengue case classification in 2009 [1]. The search was performed in three databases: PubMed, Embase, and Scopus; covering literature between the period of January 2009 and December 2018. Manual search for reference lists of included studies was performed to check additional studies relevant to the topic. The keywords used in search are “dengue” OR “severe dengue” OR “dengue severity” AND “diagnosis” OR “clinical diagnosis” OR “warning signs.” All the references were imported and removed duplicates by using bibliographical software package, EndNote version X7 (Thomas Reuters, New York, NY, USA). The studies were screened independently against the inclusion and exclusion criteria by two authors (TP and XZ), and a third author (PJ) resolved disagreement between the two reviewers regarding eligibility of a study.

### Inclusion and exclusion criteria

Studies were included if they met the following criteria: (1) any type of studies (retrospective, prospective, or cohort, case–control, cross-sectional studies) reporting severe dengue (defined with 2009 WHO diagnosis criteria) compared with dengue fever; (2) studies that distinguished clinical signs and symptoms and/or laboratory features of severe dengue and dengue fever with or without warning signs; (3) studies that published on and after 2009; (4) studies that classified dengue severity according to new 2009 WHO classification; (5) studies that included either children or adults only or both children and adults. We excluded studies if they were narrative review, letters to editors, case reports and case series, incomplete information to extract data and not written in English.

### Quality assessment

Two of the authors (TP and XZ) independently assessed the quality of each included study using the Newcastle-Ottawa Scale (NOS) [12]. NOS is the risk assessment tool developed to assess the quality of non-randomized studies used in systematic review and meta-analysis. It consists of three parameters of quality i.e. selection, comparability, and exposure with maximum of 4 points for selection of study groups, 2 points for comparability of groups and 3 points for exposures and outcomes. The NOS scores were divided into low quality (scores 1–3), intermediate quality (scores 4–6), and high-quality (scores 7–9) [13]. When any difference in opinion of quality assessment between the two authors happened, it was solved by a third author (PJ) via discussion and consensus.

### Data extraction

The data were extracted from each study through structured data extraction forms. Items extracted for the characteristics of studies included the authors, year of publication, country, setting of study, study design, study population (children, adult or both), numbers of patients for dengue fever (with or without warning signs) and severe dengue, and diagnosis of dengue. Outcome data (clinical signs and symptoms and/or laboratory features) for severe dengue and dengue fever were extracted and compiled in the summary tables by one author (HTP), and cross-checked by another author (XZ) for accuracy and relevance.

### Data analyses

Data were analyzed using RevMan software (Review Manager Version 5.3.5, The Nordic Cochrane Centre, Copenhagen). Dichotomous data was analysed using the Mantel–Haenszel (M-H) method; odds ratio (OR) with 95% conﬁdence interval (CI) was calculated using either a fixed-effect or random-effect model with at least four or more studies though only 2 studies are needed for a meta-analysis theoretically. The test of overall effect was assessed using *z*-statistics at *P* < 0.05. Heterogeneity between studies was evaluated using the Cochrane Q (*χ*^2^ test) and *I*^2^ test. *I*^2^ value considered to 0% as no, 25% as low, 50% as moderate and 75% as high heterogeneity [14]. The statistical signiﬁcance for heterogeneity was set with a *P* value < 0.10. The ﬁxed-effects model with weighting of the studies was used when there was a lack of signiﬁcant heterogeneity (*P* > 0.10), while the random-effects model with weighting of the studies was used when there was heterogeneity between studies (*P* < 0.10) [14]. Sensitivity testing to identify the effect of the subgroups was performed by subgroup analysis based on study population. Subgroup analysis was performed to (1) explore the potential sources of heterogeneity among the studies and (2) evaluate the effect in a specific subgroup. The predefined subgroups were study population (children, adult, or both) and dengue severity (severe dengue or dengue fever with or without warning signs).

## Results

### Study characteristics and quality

**Figure 2.**
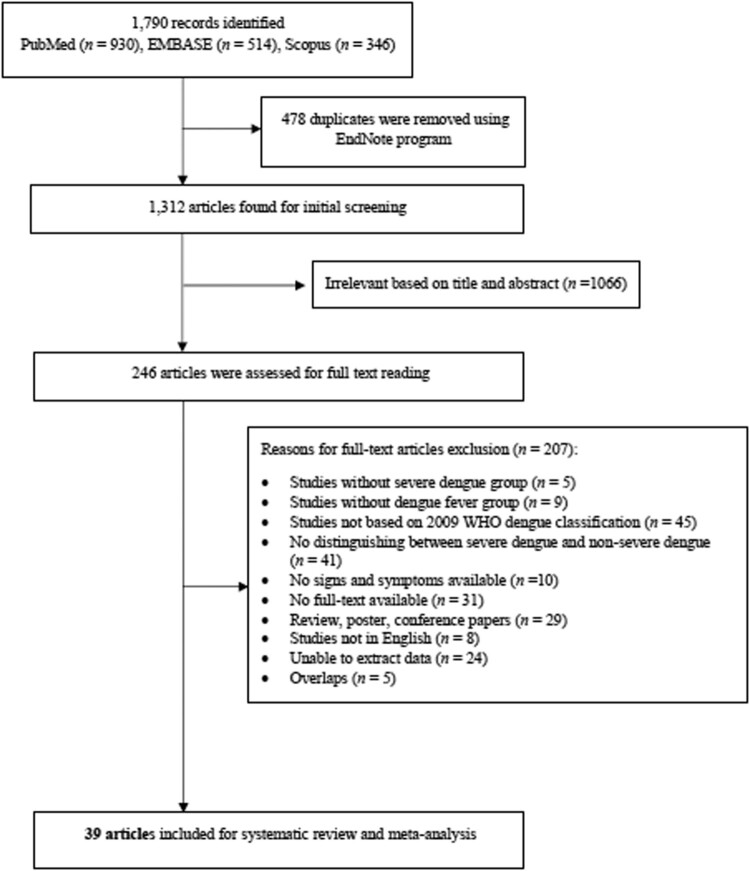
Selection of studies for inclusion in the systematic review and meta-analysis.

Figure 2 illustrates searching articles and the selection process. A total of 1790 records were identified, whereas a total of 478 duplicates were removed. The initial screening yielded 1312 articles, of which 246 articles were assessed for full text reading. A total of 207 full-text articles were excluded for the reasons mentioned in the study flow chart (Figure 2). Finally, 39 articles [2, 7–10, 15–48] were selected for inclusion in this meta-analysis according to the WHO classification for dengue, namely dengue without warning signs, dengue with warning signs and severe dengue, as well as unclassified signs or laboratory features. The date set for searching was 2009, all the studies were published after 2009.

Table 1 provides a summary characteristic of prospective study (*n* = 16), retrospective study (*n* = 21) and case control study (*n* = 2). Sample sizes were varied among the studies, ranging from 8 to 2060 cases. This study included a population of children (*n* = 18), adult (*n* = 14) and both (*n* = 7) and they are from varying locations: Asia (*n* = 31), Brazil (*n* = 4), Germany (*n* = 1), Mexico (*n* = 2), and Spain (*n* = 1). Most studies were performed in hospital settings (*n* = 36) than healthcare network (*n* = 1), medical education and research institute (*n* = 1), tertiary care unit (*n* = 1). Comorbidities were reported in ten studies, the proportion of comorbidity varied from 0% to 100% in severe dengue and 13% to 55.7% in dengue fever with or without warning signs. Nineteen studies reported day of presentation of illness or fever, whereas median day of illness ranged from 3.5 to 5 days in severe dengue and 2–5 days in dengue fever with or without warning signs. Dengue infection was confirmed by clinically in two studies, whereas serology, ELISA, PCR, HIA, viral isolation, and nucleotide detection was used together with clinical diagnosis in 37 studies for confirmation of dengue infection. Assessing the quality of the studies by Newcastle-Ottawa Scale, 5 studies were high quality (scores 7–9), 33 studies were intermediate quality (scores 4–6) and only one study has low quality (scores 1–3).

**Table 1.** Characteristics of included studies.

Author, year	Country, setting	Study design	Population	Sample size (*n*)		Comorbidity		Day of presentation (days)		Diagnosis of dengue	Quality score
				SD	DF	SD	DF	SD	DF		
Adam et al. 2018	Indonesia, hospital	Retrospective descriptive-analytic study	Children	28	112	NR	NR	4–5	4–5	Serology	4
Agarwal et al. 2018	India, hospital	Retrospective study	Children	52	136	NR	NR	NR	NR	ELISA	4
Alvarado-Castro et al. 2016	Mexico, hospital	Retrospective case series study	Children	56	77	NR	NR	5	4.6	Clinical diagnosis	5
Andries et al. 2016	Cambodia, hospital	Case-control study	Children	22	24 (without WS) 62 (with WS)	NR	NR	4	2 (without WS) 4 (with WS)	Serology, PCR, HIA	8
Athira et al. 2018	India, hospital	Retrospective cross-sectional study	Children	11	7 (without WS) 16 (with WS)	NR	NR	NR	NR	ELISA	4
Aung et al. 2013	Thailand, hospital	Retrospective study	Adult	90	193	25.6%	20.2%	4	4	PCR, Serology	5
Bhaskar et al. 2015	India, hospital	Retrospective study	Adult	128	510	13%	13%	NR	NR	ELISA	6
Carrasco et al., 2014	Singapore, hospital	Retrospective cohort study	Adult	96	500	21%	19%	3.9	4.3	PCR, Serology	6
de Cavalcanti et al. 2013	Brazil, hospital	Retrospective cross- sectional study	Both	52	4 (without WS) 28 (with WS)	NR	NR	NR	NR	ELISA, PCR, Viral isolation	5
Giraldo et al. 2011	Brazil, hospital	Retrospective cohort study	Children	30	151	23.3%	29.1%	NR	NR	Clinical diagnosis, Serology	7
Hoffmeister et al. 2015	Germany, hospital	Retrospective study	Adult	6	30 (without WS) 11 (with WS)	0%	23% (without WS) 18.1% (with WS)	NR	NR	ELISA, PCR, Serology	4
Jayaratne et al. 2012	Sri Lanka, hospital	Prospective study	Adult	40	144	NR	NR	NR	NR	ELISA, PCR	6
Kumar et al. 2014	Spain, hospital	Prospective study	Children	20	95	Exclude other febrile illness		3.5	3	ELISA	5
Lee et al. 2016	Taiwan, hospital	Retrospective study	Adult	37 (≤4 days) 18 (> 4days)	593 (≤4 days) 415 (> 4days)	54.5%	24.3%	5	3	PCR, Serology	5
Lin et al. 2016	China, hospital	Prospective study	Adult	8	130	NR	NR	NR	NR	ELISA, PCR	6
Macedo et al. 2014	Brazil, hospital	Retrospective study	Children	107	18 (without WS) 142 (with WS)	NR	NR	4	5 (without WS) 5 (with WS)	PCR, Serology	6
Michels et al. 2013	Indonesia, hospital	Prospective observational study	Adult	11	55	Exclude concurrent chronic disease and pregnancy		NR	NR	PCR, Serology	5
Nguyen et al. 2017	Vietnam, hospital	Prospective study	Children	117	1943	NR	NR	NR	NR	ELISA, PCR, Serology	7
Pereira et al. 2018	India, hospital	Retrospective study	Adult	101	449	Exclude concomitant febrile illness		NR	NR	ELISA, Serology	5
Phakhounthong et al. 2018	Cambodia, hospital	Retrospective study	Children	38	160	Exclude acquired healthcare associated infection		4.1	4.3	ELISA	5
Pozo-Aguilar et al. 2014	Mexico, hospital	Prospective cross-sectional study	Both	115 109	374 380	NR	NR	NR	NR	ELISA, PCR, Serology	7
Prasad et al. 2013	India, hospital	Prospective study	Children	45	10	NR	NR	NR	NR	ELISA, PCR	6
Ramabhatta et al. 20 17	India, hospital	Prospective cross-sectional study	Children	194	66 (without WS) 308 (with WS)	NR	NR	NR	NR	Serology	6
Rathakrishnan et al. 2014	Malaysia, hospital	Prospective descriptive study	Adult	5	64 (without WS) 388 (with WS)	NR	NR	5	5	ELISA, PCR, HIA	6
Roy et al. 2013	India, hospital	Prospective study	Children	73	15 (without WS) 32 (with WS)	Exclude concomitant infections and liver disease		NR	NR	ELISA	5
Sahana et al. 2014	India, hospital	Prospective observational study	Children	20	39 (without WS) 22 (with WS)	NR	NR	4.6	4.6	Serology	6
Singh et al. 2015	India, hospital	Prospective study	Children	17	7 (without WS) 48 (with WS)	Exclude other infections		NR	NR	ELISA, Serology	3
Soundravally et al. 2015	India, medical education and research institute	Nested case-control study	Both	20	13 (without WS) 15 (without WS)	Exclude known cases		NR	NR	ELISA, PCR	5
Sreenivasan et al. 2018	India, tertiary care center	Prospective analytical study	Children	93	266	Exclude co-infections and co-morbidities		5	5	ELISA	6
Tai et al. 2017	Australia, healthcare networks	Retrospective case series study	Both	1	123 (without WS) 84 (with WS)	100%	7.3% (without WS) 13.1% (with WS)	4	4.5 (without WS) 4 (with WS)	PCR, Serology	5
Tamibmaniam et al. 2016	Malaysia, hospital	Retrospective study	Both	59	657	22%	22%	5	5	Not reported	4
Temprasertrudee et al. 2018	Thailand, hospital	Retrospective cohort study	Adult	38	319	21.1%	14.1%	4	3	Serology	5
Thanachartwet et al. 2015	Thailand, hospital	Prospective study	Adult	216	132	Exclude mixed infection and underlying medical illness		NR	NR	ELISA, PCR	6
Thanachartwet et al. 2016	Thailand, hospital	Prospective observational study	Adult	20	105	Exclude mixed infection, underlying medical illness and pregnancy		5	4	ELISA, PCR	6
Thein et al. 2013	Singapore, hospital	Retrospective study	Both	65	248	NR	NR	4	4	PCR	4
Tsai et al. 2013	Taiwan, hospital	Retrospective study	Both	7	64 77	71.4%	6.3% (without WS) 49.4% (with WS)	4.4	3.8 (without WS) 3.6 (with WS)	PCR, Serology, HIA	4
Van de Weg et al. 2012	Indonesia, hospital	Prospective study	Children	104	69	NR	NR	4	3	PCR, Serology	6
Wakimoto et al. 2017	Brazil, hospital	Retrospective case-control study	Children	69	164	NR	NR	NR	NR	ELISA	7
Zhang et al. 2017	China, hospital	Retrospective study	Adult	38	174	Exclude chronic medical illness		NR	NR	Serology, Viral isolation, Nucleotide detection	4

Notes: DF = Dengue fever; SD = Severe dengue; ELISA = Enzyme-linked immunosorbent assay; PCR = Polymerase chain reaction; HIA = Hemagglutination inhibition assay; NR = Not reported; WS = Warning signs; Day of presentation = day of illness or fever prior to admission/first contact with health services

### Potential predictive factors of severe dengue

**Figure 3.**
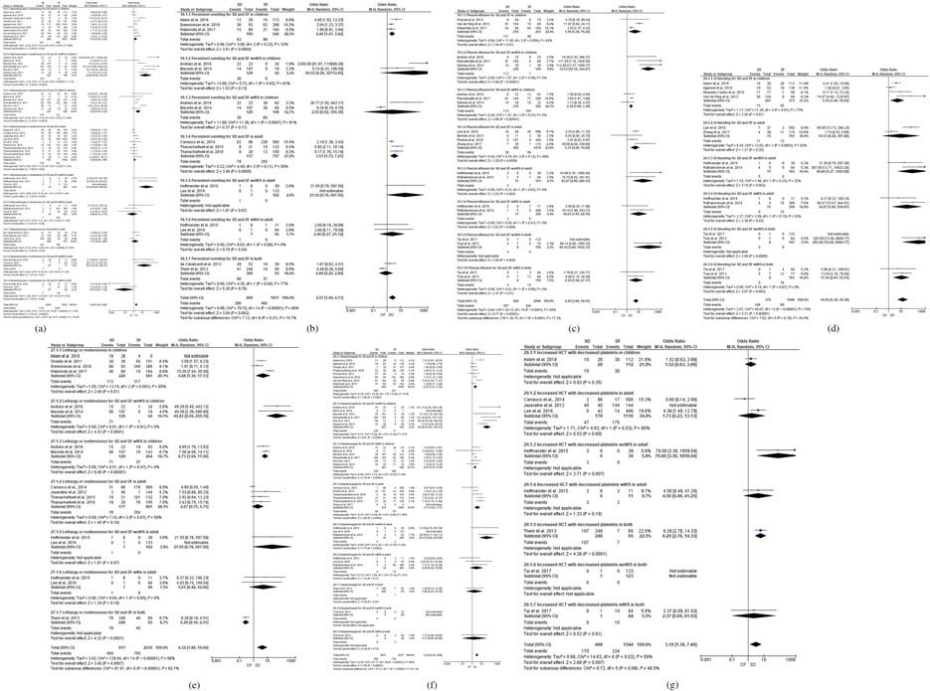
Forest plots comparison of signs and symptoms for severe dengue and dengue fever.

A total of 39 factors were analyzed when there are four or more studies to perform a regression analysis (Table 2; Figure 3). The fixed effect model was used in 12 factors (nausea, headache, retro-orbital pain, arthralgia, myalgia, hematuria, cough, diarrhea, splenomegaly, shock, dyspnea, gall bladder wall thickening), while the random effect model was used in 27 factors (gender: male and female, comorbidity, fever, vomiting, rash, tourniquet test (+), leucopenia, abdominal pain or tenderness, persistent vomiting, pleural effusion, ascites, epistaxis, gum bleeding, gastrointestinal bleeding (hematemesis and/or melena), vaginal bleeding, lethargy or restlessness, hepatomegaly > 2 cm, increased HCT with decreased platelets, skin bleeding (petechiae, purpura, ecchymosis), impaired consciousness, thrombocytopenia (platelets < 150*10^9^/L), elevated ALT (>40 u/l), elevated AST (>40 u/l), hypoalbuminemia, primary infection, secondary infection). Of these factors, a total of 21 factors were found to be significantly associated with severe dengue and dengue fever with or without warning signs.

**Table 2.** Results of meta-analysis for the clinical characteristics between severe dengue and dengue fever with or without warning signs.

Clinical characteristics	Number of studies	Total events	Odds ratio	Z	Test for OR	Test of heterogeneity		Model
		SD/DF	(95% CI)		*P*-value	*I*^2^ (%)	*P*-value	
*Demographic characteristics*								
Gender (Male)	22	584/2483	0.95 (0.77–1.16)	0.53	0.60	33	0.04	Random
Gender (Female)	17	485/1199	1.30 (0.95–1.77)	1.66	0.10	62	<0.001	Random
Comorbidity	8	100/545	2.03 (1.09–3.78)	2.24	0.03	70	<0.001	Random
*Probable dengue*								
Fever	14	951/3076	0.74 (0.34–1.60)	0.77	0.44	52	0.01	Random
Nausea	8	140/461	0.92 (0.66–1.27)	0.53	0.60	13	0.32	Fixed
Vomiting	19	849/2275	2.18 (1.50–3.16)	4.12	<0.001	77	<0.001	Random
Rash	22	395/1569	1.07 (0.84–1.37)	0.55	0.58	41	0.01	Random
Headache	18	505/2388	0.84 (0.70–1.00)	2.00	0.05	28	0.11	Fixed
Retro-orbital pain	13	172/726	0.99 (0.75–1.30)	0.10	0.92	0	0.73	Fixed
Arthralgia	16	281/1566	1.10 (0.89–1.36)	0.86	0.39	0	0.55	Fixed
Myalgia	17	451/2498	1.01 (0.83–1.24)	0.11	0.92	0	0.53	Fixed
Tourniquet test (+)	7	108/349	0.52 (0.19–1.44)	1.27	0.21	68	<0.01	Random
Leucopenia	14	275/1578	0.82 (0.59–1.15)	1.15	0.25	35	0.06	Random
*Warning signs*								
Abdominal pain or tenderness	33	1338/2554	2.00 (1.49–2.68)	4.62	<0.001	75	<0.001	Random
Persistent vomiting	12	296/465	2.57 (1.40–4.73)	3.04	0.002	80	<0.001	Random
Clinical fluid accumulation								
Pleural effusion	14	397/264	6.20 (3.66–10.51)	6.77	<0.001	65	<0.001	Random
Ascites	15	420/266	5.20 (3.27–8.29)	6.94	<0.001	54	0.002	Random
Gall bladder wall thickening	4	141/80	5.61 (2.73–11.53)	4.69	<0.001	31	0.19	Fixed
Mucosal bleeding								
Epistaxis	9	73/110	2.23 (1.04–4.77)	2.07	0.04	65	0.001	Random
Gum bleeding	10	48/208	3.34 (1.60–6.98)	3.21	<0.01	49	0.02	Random
GI bleeding (hematemesis and/or melena)	10	104/89	14.56 (5.38–39.39)	5.27	<0.001	74	<0.001	Random
Hematuria	4	4/22	2.48 (0.75–8.25)	1.48	0.14	0	0.53	Fixed
Vaginal bleeding	4	20/21	6.62 (0.38–114.64)	1.30	0.19	75	<0.01	Random
Skin bleeding (petechiae, purpura, ecchymosis)	19	386/723	2.12 (1.53–3.19)	4.22	<0.001	62	<0.001	Random
Lethargy or restlessness	13	464/755	4.32 (1.86–10.04)	3.40	<0.001	89	<0.001	Random
Hepatomegaly > 2 cm	25	796/730	3.34 (2.38–4.68)	7.00	<0.001	66	<0.001	Random
Increased HCT with decreased platelets	7	170/224	3.19 (1.36–7.46)	2.68	0.007	59	0.02	Random
*Severe dengue*								
Severe plasma leakage								
Shock	6	235/3	47.51 (14.80 -152.50)	8.85	<0.001	35	0.15	Fixed
Dyspnea	6	99/44	11.19 (6.91–18.11)	9.82	<0.001	0	0.56	Fixed
Severe organ involvement								
Elevated ALT (>40 u/L)	7	290/582	3.24 (1.87–5.61)	4.19	<0.001	51	0.04	Random
Elevated AST (>40 u/L)	8	338/790	3.75 (2.11–6.68)	4.49	<0.001	51	0.03	Random
Impaired consciousness	5	37/30	29.81 (4.08–217.94)	3.34	<0.001	74	0.002	Random
Splenomegaly	6	34/75	1.33 (0.81–2.18)	1.14	0.25	0	0.76	Fixed
*Others*								
Cough	6	36/398	1.08 (0.73–1.59)	0.39	0.70	0	0.60	Fixed
Diarrhea	12	71/704	1.02 (0.76–1.36)	0.13	0.89	0	0.99	Fixed
Thrombocytopenia (platelets <150*109/L)	18	893/3282	2.70 (1.60–4.55)	3.73	<0.001	68	<0.001	Random
Hypoalbuminemia	7	152/776	2.25 (0.85–5.92)	1.64	0.10	78	<0.001	Random
Primary infection	4	11/83	0.43 (0.09–2.04)	1.07	0.29	64	0.03	Random
Secondary infection	5	96/310	1.93 (1.25–2.97)	2.96	0.003	0	0.50	Random

Notes: SD = Severe dengue; DF = Dengue fever; HCT = Hematocrit; ALT = Alanine transaminase; AST = Aspartate transaminase.

### Socio-demographic characteristics

Socio-demographic characteristics including gender difference (male and female) showed no significant association with severe dengue (*P *> 0.05). Pooling of eight studies, comorbidity was positively associated with severe dengue (OR: 2.03, CI: 1.09–3.78, *z* = 2.24, *P *= 0.03).

### Probable dengue without warning signs

The symptoms listed for probable dengue without warning signs include fever, nausea, vomiting, rash, headache, retro-orbital pain, arthralgia, myalgia, positive tourniquet test and leucopenia. Amongst all listed symptoms, vomiting was positively associated with severe dengue (OR: 2.18, CI: 1.50–3.16, *z* = 4.12, *P *< 0.001) in 19 studies.

### Dengue with warning signs

The symptoms listed for dengue with warning signs include abdominal pain or tenderness, persistent vomiting, clinical fluid accumulation (pleural effusion, ascites, gallbladder wall thickening), mucosal bleeding (epistaxis, gum bleeding, gastrointestinal bleeding, hematuria, vaginal bleeding, skin bleeding), lethargy or restlessness, hepatomegaly >2 cm and increased hematocrit with decreased platelets. Of the listed symptoms, pleural effusion (OR: 6.20, CI: 3.66–10.51, *z* = 6.77, *P *< 0.001), ascites (OR: 5.20, CI: 3.27–8.29, *z* = 6.94, *P *< 0.001), gallbladder wall thickening (OR: 5.61, CI: 2.73–11.53, *z* = 4.69, *P *< 0.001), and gastrointestinal bleeding as a manifestation of mucosal bleeding (OR: 14.56, CI: 5.38–39.39, *z* = 5.27, *P *< 0.001) were highly associated with severe dengue for a patient being diagnosed with dengue with warning signs. In addition, of the warning signs, abdominal pain or tenderness (OR: 2.00, CI: 1.49–2.68, *z* = 4.62, *P *< 0.001), persistent vomiting (OR: 2.57, CI: 1.40–4.73, *z* = 3.04, *P *= 0.002), epistaxis (OR: 2.23, CI: 1.04–4.77, *z* = 2.07, *P *= 0.04), gum bleeding (OR: 3.34, CI: 1.60–6.98, *z* = 3.21, *P *< 0.01), skin bleeding (OR: 2.12, CI: 1.53–3.19, *z* = 4.22, *P *< 0.001), lethargy or restlessness (OR: 4.32, CI: 1.86–10.04, *z* = 3.40, *P *< 0.001), hepatomegaly >2 cm (OR: 3.34, CI: 2.38–4.68, *z* = 7.00, *P *< 0.001) and raising hematocrit (OR: 3.19, CI: 1.36–7.46, *z* = 2.68, *P *= 0.007) were moderately associated with severe dengue.

### Severe dengue

The symptoms listed for severe dengue include shock, fluid accumulation leading to dyspnea, severe bleeding on clinical evaluation, impaired consciousness and transaminitis (aspartate aminotransferase (AST) or alanine aminotransferase (ALT) ≥1000 units/L) and organ failure. Symptoms of shock (OR: 47.51, CI: 14.80–152.50, *z* = 8.85, *P *< 0.001), dyspnea (OR: 11.19, CI: 6.91–18.11, *z* = 9.82, *P *< 0.001) and impaired consciousness (OR: 29.81, CI: 4.08–217.94, *z* = 3.34, *P *< 0.001) had remarkably higher odds for severe dengue. Elevated ALT (OR: 3.24, CI: 1.87–5.61, *z* = 4.19, *P *< 0.001), elevated AST (OR: 3.75, CI: 2.11–6.68, *z* = 4.49*, P* < 0.001) were moderately associated with severe dengue.

### Other signs and symptoms and laboratory features

Other symptoms of cough and diarrhoea in association with dengue infection were analysed but yielded non-significant results. Associated laboratory features of thrombocytopenia (OR: 2.70, CI: 1.60–4.55, *z* = 3.73, *P *< 0.001) was positively associated with severe dengue while hypoalbuminemia found no association with severe dengue (*P *> 0.05). The presence of a secondary dengue infection (a patient having a second or more dengue infection) was also statistically significant in the odds of being diagnosed with dengue infection (OR: 1.93, CI: 1.25–2.97, *z* = 2.96, *P *< 0.01).

## Discussion

Our detailed meta-analysis comprises studies encompassing numerous countries globally suggests the current 2009 WHO clinical classification optimally identifies severe dengue infection.

Our study lies in its detailed meta-analysis of a wide range of studies encompassing numerous countries globally. We found that patients with comorbidity had 2-times higher risk of progression into severe dengue. This finding is in line with previous study indicating that pre-existing comorbidities were risk factors of severe organ involvement in dengue patients [49]. Digestive factors of vomiting, persistent vomiting, abdominal pain or tenderness were indicative of severe dengue in our study, which is consistent with previous study showing that vomiting and abdominal pain were most prevalent warning signs which occur prior to severe dengue [50]. Bleeding manifestations include mucosal bleeding (epistaxis, gum bleeding), GI bleeding (hematemesis and/or melena) and skin bleeding (petechiae, purpura, ecchymosis) were shown as valuable predictors of severe dengue in our study except for hematuria and vaginal bleeding. Consistent with previous meta-analyses, four kinds of bleeding: epistaxis, gum bleeding, hematemesis, and melena were related to the risk of development of patients with severe dengue [13]. Among bleeding factors, gastrointestinal bleeding proved highly indicative of severe dengue. A study also showed that patients with gastrointestinal bleeding had the highest risk of progressing into severe disease [23]. Notably, pleural effusion and ascites were significantly associated with severe dengue. Plasma leakage causing fluid accumulation, during which fever transitions into defervesence, was cited as a critical indicator of progression to severe dengue [33]. Concurrent increase in haemotocrit and rapid decrease in platelet count, vomiting and abdominal distention were significant in predicting the likelihood of severe plasma leakage as a warning sign of dengue [22]. In one Singaporean study, concurrent increase in haemotocrit and decrease in platelet count were found to be predictive of severe haemorrhage [22], which is consistent with our result.

Liver damage is a common complication of dengue, liver enzymes are valuable markers during dengue infection [51]. In our results, hepatomeagly (>2 cm), elevated AST and ALT were significantly different between severe dengue and dengue with or without warning signs. These findings are similar to the previous studies that liver enlargement and liver enzymes (AST and ALT) were significantly higher in severe dengue patient [48, 52]. Interestingly, four articles highlighted the presence of gallbladder wall thickening as a clinical sign of dengue infection and which was found to be associated with severe dengue. In multiple studies, this was characteristic only for severe dengue [25, 53]. One study showed that gallbladder thickening was present even before serological tests were positive [54] and as potential early predictors [53]. While thrombocytopenia was a significant predictor for severe dengue in many studies [52, 55], our result revealed that platelet count less than 150000/mm^3^ has value in ruling in dengue infection. However, one study surprisingly showed that it was unlikely to be a direct precipitant for clinical manifestations of bleeding [38]. Our analysis showed association of secondary dengue infection with severe dengue. As proven by other studies, patients presenting with a secondary dengue infection were associated with a higher risk of developing severe dengue [33, 56], which suggests that the clinical presentation of severe dengue was affected by both host factors (secondary immune response and viral load) [57].

Our review has several limitations. Firstly, there was variability among the included studies in terms of study designs, study population, diagnoses, comorbidities and day of presentation of illness or fever, which weakens the comparison among different studies. The definitions and cutoff values of warning signs and severity were widely varied within the studies [58], which brought heterogenous application to rule out the cases. Some identified studies were performed on individuals of one demographic, such as being either from the paediatric or adult age group, which can lead to unaccounted variation in presenting signs or symptoms. Secondly, research conducted in regions endemic for dengue infection, especially countries near the equator, constituted an overwhelming majority in our study. Therefore, studies of dengue infection in less endemic countries could have been elided over, conferring selection bias for our study. Thirdly, in our meta-analysis for dengue without warning signs, it was unfortunate that a majority of the listed symptoms did not prove significant. Many listed symptoms that we studied also did not stem from an acceptable level of heterogeneity.

Our finding identified significant association between 21 factors (comorbidity, vomiting, persistent vomiting, abdominal pain or tenderness, pleural effusion, ascites, epistaxis, gum bleeding, GI bleeding, skin bleeding, lethargy or restlessness, hepatomegaly (>2 cm), increased HCT with decreased platelets, shock, dyspnea, impaired consciousness, thrombocytopenia, elevated AST and ALT, gall bladder wall thickening and secondary infection) and severe dengue.

Therefore, these clinical signs and symptoms may be useful for triaging potential severe dengue in patients and may further guide further enhancement of the current WHO dengue severity classifications, though heterogenicity was considerably high. More large-scale multicenter studies may be carried on identifying the association of with severe dengue using standard definitions and classification.

## Supplementary Material

S2_Quality_assessment.docx

S1_Indexed_and_keyword_terms.docx

## Geolocation information

Asia, Europe, America.

## Appendices

### Appendix 1. Indexed and keyword terms for searching in three databases

**Table:**

Databases	Indexed and keyword terms
Pubmed	((((“Dengue”[Mesh]) OR dengue)) AND (((“Severe Dengue”[Mesh]) OR severe dengue) OR dengue severity)) AND (((“Diagnosis”[Mesh]) OR clinical diagnosis) OR warning signs) Filters: Publication date from 2009/01/01 to 2018/12/31
Embase	((“dengue”/exp OR “dengue”) AND “severe dengue”/exp OR “severe dengue” OR “dengue severity”) AND (“diagnosis”/exp OR “diagnosis” OR “clinical diagnosis” OR “warning signs”) AND (2009–2018)
Scopus	(TITLE-ABS-KEY (dengue AND severe AND dengue)) AND (diagnosis OR warning AND signs) (2009–2018)

### Appendix 2. Quality assessment of studies using Newcastle-Ottawa scale.

**Table:**

Author, Year	Selection				Comparability	Outcome			Total
	Representativeness of the exposed cohort or case	Selection of the non-exposed cohort or control	Ascertainment of exposure or adequate case definition	Outcome of interest was not present at the start of studyor definit ion of control		Assessment of outcome or exposure	Follow up long enough for outcomes to occur or ascertainment for case and control	Adequacy of follow up or non-response rate	
Adam et al. 2018	0	1	1	0	0	1	0	1	4
Agarwal et al. 2018	0	1	1	0	0	1	0	1	4
Alvarado- Castro et al. 2016	0	1	1	0	1	1	0	1	5
Andries et al. 2016	1	1	1	1	2	1	1	0	8
Athira et al. 2018	0	1	1	0	0	1	0	1	4
Aung et al. 2013	0	1	1	0	1	1	0	1	5
Bhaskar et al. 2015	0	1	1	0	2	1	0	1	6
Carrasco et al., 2014	0	1	1	0	2	1	0	1	6
de Cavalcanti et al. 2013	1	1	1	0	0	1	0	1	5
Giraldo et al. 2011	1	1	1	0	2	1	0	1	7
Hoffmeister et al. 2015	0	1	1	0	0	1	0	1	4
Jayaratne et al. 2012	0	1	1	1	0	1	1	1	6
Kumar et al. 2014	0	1	1	1	0	1	1	0	5
Lee et al. 2016	0	1	1	0	1	1	0	1	5
Lin et al. 2016	0	1	1	1	0	1	1	1	6
Macedo et al. 2014	1	1	1	0	1	1	0	1	6
Michels et al. 2013	0	1	1	1	0	1	1	0	5
Nguyen et al. 2017	1	1	1	1	1	1	1	0	7
Pereira et al. 2018	0	1	1	0	1	1	0	1	5
Phakhounth ong et al. 2018	0	1	1	0	1	1	0	1	5
Pozo- Aguilar et al. 2014	1	1	1	0	1	1	1	1	7
Prasad et al. 2013	0	1	1	1	0	1	1	1	6
Ramabhatta et al. 20 17	0	1	1	1	0	1	1	1	6
Rathakrishn an et al. 2014	0	1	1	1	0	1	1	1	6
Roy et al. 2013	0	1	1	0	0	1	1	1	5
Sahana et al. 2014	0	1	1	0	1	1	1	1	6
Singh et al. 2015	0	1	1	0	0	1	0	0	3
Soundravall y et al. 2015	0	0	1	1	0	1	1	1	5
Sreenivasan et al. 2018	0	1	1	0	1	1	1	1	6
Tai et al. 2017	1	1	1	0	0	1	0	1	5
Tamibmani am et al. 2016	0	1	1	0	1	1	0	0	4
Temprasertr udee et al. 2018	0	1	1	0	1	1	0	1	5
Thanachart wet et al. 2015	0	1	1	0	1	1	1	1	6
Thanachart wet et al. 2016	0	1	1	0	1	1	1	1	6
Thein et al. 2013	0	1	1	0	0	1	0	1	4
Tsai et al. 2013	0	1	1	0	0	1	0	1	4
Van de Weg et al. 2012	0	1	1	1	0	1	1	1	6
Wakimoto et al. 2017	1	0	1	1	1	1	1	1	7
Zhang et al. 2017	0	1	1	0	0	1	0	1	4
