# Supplementary material for: Clinical signs and symptoms associated with WHO severe dengue classification: a systematic review and meta-analysis
Source: Emerg Microbes Infect. 2021 Jun 11;10(1):1116–28. doi: 10.1080/22221751.2021.1935327 (PMC8205005; doi:10.1080/22221751.2021.1935327)
Supplement: S2_Quality_assessment.docx [file TEMI_A_1935327_SM2155.docx]

**Appendix 2**. Quality assessment of studies using Newcastle-Ottawa scale

| Author, Year | Selection | | | | Comparability | Outcome | | | Total |
| --- | --- | --- | --- | --- | --- | --- | --- | --- | --- |
|  | Representativeness of the exposed cohort or case | Selection of the non-exposed cohort or control | Ascertainment of exposure or adequate case definition | Outcome of interest was not present at the start of study or definition of control |  | Assessment of outcome or exposure | Follow up long enough for outcomes to occur or ascertainment for case and control | Adequacy of follow up or non-response rate |  |
| Adam et al. 2018 | 0 | 1 | 1 | 0 | 0 | 1 | 0 | 1 | 4 |
| Agarwal et al. 2018 | 0 | 1 | 1 | 0 | 0 | 1 | 0 | 1 | 4 |
| Alvarado-Castro et al. 2016 | 0 | 1 | 1 | 0 | 1 | 1 | 0 | 1 | 5 |
| Andries et al. 2016 | 1 | 1 | 1 | 1 | 2 | 1 | 1 | 0 | 8 |
| Athira et al. 2018 | 0 | 1 | 1 | 0 | 0 | 1 | 0 | 1 | 4 |
| Aung et al. 2013 | 0 | 1 | 1 | 0 | 1 | 1 | 0 | 1 | 5 |
| Bhaskar et al. 2015 | 0 | 1 | 1 | 0 | 2 | 1 | 0 | 1 | 6 |
| Carrasco et al., 2014 | 0 | 1 | 1 | 0 | 2 | 1 | 0 | 1 | 6 |
| de Cavalcanti et al. 2013 | 1 | 1 | 1 | 0 | 0 | 1 | 0 | 1 | 5 |
| Giraldo et al. 2011 | 1 | 1 | 1 | 0 | 2 | 1 | 0 | 1 | 7 |
| Hoffmeister et al. 2015 | 0 | 1 | 1 | 0 | 0 | 1 | 0 | 1 | 4 |
| Jayaratne et al. 2012 | 0 | 1 | 1 | 1 | 0 | 1 | 1 | 1 | 6 |
| Kumar et al. 2014 | 0 | 1 | 1 | 1 | 0 | 1 | 1 | 0 | 5 |
| Lee et al. 2016 | 0 | 1 | 1 | 0 | 1 | 1 | 0 | 1 | 5 |
| Lin et al. 2016 | 0 | 1 | 1 | 1 | 0 | 1 | 1 | 1 | 6 |
| Macedo et al. 2014 | 1 | 1 | 1 | 0 | 1 | 1 | 0 | 1 | 6 |
| Michels et al. 2013 | 0 | 1 | 1 | 1 | 0 | 1 | 1 | 0 | 5 |
| Nguyen et al. 2017 | 1 | 1 | 1 | 1 | 1 | 1 | 1 | 0 | 7 |
| Pereira et al. 2018 | 0 | 1 | 1 | 0 | 1 | 1 | 0 | 1 | 5 |
| Phakhounthong et al. 2018 | 0 | 1 | 1 | 0 | 1 | 1 | 0 | 1 | 5 |
| Pozo-Aguilar et al. 2014 | 1 | 1 | 1 | 0 | 1 | 1 | 1 | 1 | 7 |
| Prasad et al. 2013 | 0 | 1 | 1 | 1 | 0 | 1 | 1 | 1 | 6 |
| Ramabhatta et al. 20 17 | 0 | 1 | 1 | 1 | 0 | 1 | 1 | 1 | 6 |
| Rathakrishnan et al. 2014 | 0 | 1 | 1 | 1 | 0 | 1 | 1 | 1 | 6 |
| Roy et al. 2013 | 0 | 1 | 1 | 0 | 0 | 1 | 1 | 1 | 5 |
| Sahana et al. 2014 | 0 | 1 | 1 | 0 | 1 | 1 | 1 | 1 | 6 |
| Singh et al. 2015 | 0 | 1 | 1 | 0 | 0 | 1 | 0 | 0 | 3 |
| Soundravally et al. 2015 | 0 | 0 | 1 | 1 | 0 | 1 | 1 | 1 | 5 |
| Sreenivasan et al. 2018 | 0 | 1 | 1 | 0 | 1 | 1 | 1 | 1 | 6 |
| Tai et al. 2017 | 1 | 1 | 1 | 0 | 0 | 1 | 0 | 1 | 5 |
| Tamibmaniam et al. 2016 | 0 | 1 | 1 | 0 | 1 | 1 | 0 | 0 | 4 |
| Temprasertrudee et al. 2018 | 0 | 1 | 1 | 0 | 1 | 1 | 0 | 1 | 5 |
| Thanachartwet et al. 2015 | 0 | 1 | 1 | 0 | 1 | 1 | 1 | 1 | 6 |
| Thanachartwet et al. 2016 | 0 | 1 | 1 | 0 | 1 | 1 | 1 | 1 | 6 |
| Thein et al. 2013 | 0 | 1 | 1 | 0 | 0 | 1 | 0 | 1 | 4 |
| Tsai et al. 2013 | 0 | 1 | 1 | 0 | 0 | 1 | 0 | 1 | 4 |
| Van de Weg et al. 2012 | 0 | 1 | 1 | 1 | 0 | 1 | 1 | 1 | 6 |
| Wakimoto et al. 2017 | 1 | 0 | 1 | 1 | 1 | 1 | 1 | 1 | 7 |
| Zhang et al. 2017 | 0 | 1 | 1 | 0 | 0 | 1 | 0 | 1 | 4 |
