# Supplementary material for: Clinical signs and symptoms associated with WHO severe dengue classification: a systematic review and meta-analysis
Source: Emerg Microbes Infect. 2021 Jun 11;10(1):1116–28. doi: 10.1080/22221751.2021.1935327 (PMC8205005; doi:10.1080/22221751.2021.1935327)
Supplement: S1_Indexed_and_keyword_terms.docx [file TEMI_A_1935327_SM2154.docx]

**Appendix 1.** Indexed and keyword terms for searching in three databases

| Databases | Indexed and keyword terms |
| --- | --- |
| Pubmed | (((("Dengue"[Mesh]) OR dengue)) AND ((("Severe Dengue"[Mesh]) OR severe dengue) OR dengue severity)) AND ((("Diagnosis"[Mesh]) OR clinical diagnosis) OR warning signs) Filters: Publication date from 2009/01/01 to 2018/12/31 |
| Embase | (('dengue'/exp OR ‘dengue’) AND  'severe dengue'/exp OR 'severe dengue' OR 'dengue severity') AND ('diagnosis'/exp OR ‘diagnosis’ OR ‘clinical diagnosis’ OR ‘warning signs’) AND (2009-2018) |
| Scopus | (TITLE-ABS-KEY (dengue AND severe AND dengue)) AND (diagnosis OR warning AND signs) (2009-2018) |
